# Removal of One-Half of the Inferior Maxillary for Osteo-Sarcoma

**Published:** 1869-10

**Authors:** S. R. Beckwith


### ARTICLE XI.

### *Removal of One-Half of the Inferior Maxillary for Osteo-Sarcoma.*

By S. R. Beckwith, M.D.

Mrs. B——, aged thirty-six, observed, some three years before I became acquainted with her case, a small tumor on the outer portion of the lower jaw, about mid-way between the symphysis and angle. It soon increased in size; at times was painful. Her physician was consulted, and treated her for six months. During his treatment the growth was slowly yet steadily increasing, until he advised removal as the only successful treatment. She would not consent, and did not again apply for treatment, preferring to let it grow uninterrupted, until it had filled the cavity of the mouth and very largely distended the cheek. For months she had not been able to take any solid food, from inability to masticate, and want of room for the introduction of food. Her nourishment was fluids taken through a tube. She had become excessively emaciated, and fearing that she soon would die, was induced to visit me. From the absence of any marked constitutional disturbance during its early growth, and its large size, the sensation of firmness being less than in exostosis or osteoma, with the peculiar crackling sensation noticed when firmly pressed upon, gave me no difficulty in diagnosing it to be a case of osteo-sarcoma, and I advised resection and disarticulation of one-half of the lower jaw. She readily consented, as her mind had been made up before leaving home that an operation would be necessary, and came prepared.

She was taken before the class, and with the assistance of Drs. Morrill and Bissell, it was removed in the following manner: The patient was seated in a chair, with her head thrown back, and held by an assistant. After the administering of chloroform, an incision was made along the base of the jaw, from the angle to a short distance beyond the symphysis, and a vertical cut extending from the termination of the first to the angle of the lips. The flaps were dissected above and below, freely exposing the tumor. The jaw was divided about one inch from the centre, toward the sound side, as the disease had extended beyond the symphysis. The patient was now allowed to become conscious, for fear of strangulation, by blood flowing into the trachea during the balance of the operation.

Her strength was very much exhausted, and we gave her freely of brandy, and allowed her to lie down for a short time, until she expressed a desire to have us proceed. A cord was fastened around the jaw, and forcibly drawing it outwards, the attachments were divided, with a strong scalpel, to the angle. The bone was now drawn outwards and forwards, putting its upper attachments on the stretch as much as possible. A bistoury was inserted behind the coronoid process, just below the zygomatic arch, and the temporal muscle was divided at its insertion. The jaw was now depressed, and disarticulating the condyloid process, it was drawn forward with force, so as to remove it from the artery. The capsular ligament and pterygoid muscles were separated, and the jaw was freed entirely, save some slight attachments of the mouth, near the angle, that had not been reached before. Very little blood was lost during the operation, and but few ligatures applied to bleeding vessels. The wound was closed by a few wire sutures, and straps applied between them; pledgets of lint, wet with a weak solution of *Calendula*, were applied to the wonnd, and held in position by simply a handkerchief.

The patient remained under our care three weeks, and then returned home. The wound healed almost its entire course by the first intention, and there were no untoward symptoms during the convalesence. Great care and attention were given the patient for the first few days after the operation, to prevent sinking, although she had no symptoms of dangerous exhaustion, but these were feared from her feeble condition prior to the resection.

Three years after the operation I saw the patient, and the deformity was not as great as I had anticipated. In place of the bone, a fibrinous deposit had filled up the gap in such a manner as to tolerably well preserve the contour of the face, and she was in good health.—*Med. Investigator*.

